# Workforce strategies during the first wave of the COVID-19 pandemic: a retrospective online survey at intensive care units in Germany

**DOI:** 10.1186/s12913-024-10848-w

**Published:** 2024-04-01

**Authors:** Lara C. Stroth, Franziska Jahns, Berit Bode, Maike Stender, Michelle Schmidt, Heiko Baschnegger, Nurith Epstein, Benedikt Sandmeyer, Carla Nau

**Affiliations:** 1 Department of Anaesthesiology and Intensive Care, University Medical Centre Schleswig-Holstein, Campus Lübeck, Ratzeburger Allee 160, 23538 Lübeck, Germany; 2 Department of Anaesthesiology, Medical Faculty, University Hospital RWTH Aachen, Pauwelsstraße 30, 52074 Aachen, Germany; 3 AIXTRA-Competence Center for Training and Patient Safety, Medical Faculty, RWTH Aachen, Forckenbeckstraße 71, 52074 Aachen, Germany; 4 Institut für Notfallmedizin und Medizinmanagement (INM), Klinikum der Universität München, LMU München, Schillerstr. 53, 80336 Munich, Germany; 5 Institute for Medical Education, University Hospital, LMU Munich, Ziemssenstraße 5, 80336 Munich, Germany

**Keywords:** Recruitment, COVID-19 pandemic, ICU staff, ICU-Response survey, Intensive care unit personnel, Staff reallocation, Workforce planning, Organizational resilience

## Abstract

**Background:**

As the COVID-19 pandemic swept across the globe at the beginning of 2020, healthcare systems were forced to rapidly adapt and expand to meet the sudden surge in demand for intensive care services. This study is the first systematic analysis of the strategies employed by German hospitals to recruit personnel and expand bed capacities during the first wave of the pandemic, and to evaluate the effectiveness of those recruitment measures.

**Methods:**

152 German hospitals with intensive care capacities were selected and invited to participate in an online-based retrospective survey. Factors like the geographic distribution, individual COVID burden and level of care were considered for inclusion in the sample. The data were analyzed descriptively.

**Results:**

A total of 41 hospitals participated in the survey. The additional demand for intensive care beds was met primarily by activating intensive care beds that were previously considered as non-operational in existing intensive care units (81% of respondents) and by upgrading recovery rooms (73%). The physician staffing requirements were met at approximately 75%, while the nursing staffing requirements were only met by about 45%. Staffing needs were met through reallocations/transfers (85%), staff recruitment from parental leave or retirement (49%), increased hours worked by internal staff (49%), new staff hiring (44%) and increased use of temporary staff (32%). Staff reallocations/transfers to critical care within a hospital were rated as the most effective measure. In this context, specialized personnel mostly from anesthesiology departments were appointed to intensive care medicine.

**Conclusions:**

Despite multiple recruitment efforts, the pandemic has exacerbated the nursing staff shortage. The reallocation of existing staff within hospitals was a key element in covering the staffing needs. However, additional measures and efforts are required in order to ensure that critically ill patients can be cared for without compromise. The results of this study may have important implications for healthcare providers and policymakers, offering an evidence-based foundation for responding to future public health emergencies with agility, efficiency, and success.

**Supplementary Information:**

The online version contains supplementary material available at 10.1186/s12913-024-10848-w.

## Background

The COVID-19 pandemic posed substantial challenges to the world and Germany in early 2020. Expanding intensive care capacities and recruiting additional staff were two key issues for hospitals in coping with the crisis.

In Germany, the first case of a SARS-CoV-2 infection was reported at the end of January 2020 [1]. The first larger outbreaks then occurred as a result of local festivities (e.g., carnival) in mid-February, resulting in increased numbers of cases in individual counties (e.g., Heinsberg) and initiating the onset of the first COVID-19 wave (calendar week 10/2020–20/2020) [2]. As a result of the infection incidence worldwide, the World Health Organization (WHO) declared the outbreak a pandemic on March 11, 2020.

In view of the expected increase in the number of infections, there was an urgent need to expand technical and personnel intensive care capacities as quickly as possible. The present study seeks to determine the strategies that were used in German hospitals and to rate their effectiveness.

Due to the dynamics of the infectious event and the novel disease that hospitals were confronted with, the first wave is to be considered as particularly critical and required hospitals to quickly and constantly adjust their resources. The pandemic had a severe impact on staff capacities in all German hospitals and necessitated the mobilization of additional medical and nursing staff on a short-term (and temporary) basis to ensure/maintain the care of critically ill patients. This was particularly challenging due to the shortage of nursing staff that existed even before the pandemic. Approximately 28,000 specialists (2019) in geriatric, health and nursing care were lacking [3]. In intensive care, 53% of hospitals reported to have staffing problems (as of fall 2016), with an average of 4.7 full-time positions not being filled [4]. A total of 3,150 full-time positions in intensive care remained vacant nationwide with an increasing tendency. Hospitals with staffing problems in the medical service of their intensive care units (ICUs) (29%) had a mean of 1.7 full-time positions vacant. Understaffing of nursing staff in ICUs can have serious consequences for patient care and is associated with an increased risk of mortality, hospital-related infections and pressure wounds [5, 6]. Due to lack of child care, staff’s own illness, or quarantine, the pandemic led to additional staff absences. Consequently, alternative concepts for staff recruitment (and training) needed to be developed.

In the context of extending intensive care capacities, the Federal Ministry of Health addressed hospitals in an open letter on March 13, 2020, asking them to recruit additional staff. It also appealed to postpone scheduled surgeries and interventions to build up additional provisional bed and treatment capacities [7]. At the same time, hospitals were assured of financial compensation for the resulting additional economic burden. In addition, a bonus was introduced for each additional intensive care bed provisionally placed or kept available. The relevant legal framework for this was laid down in the COVID-19 Hospital Relief Act (*COVID-19-Krankenhausentlastungsgesetz*), which came into force on March 27, 2020. Even before the pandemic, Germany had a very high density by international standards, with 33.9 intensive care beds per 100,000 inhabitants [8]. Capacities were significantly lower, for example, in Spain and Italy, which were severely affected at the beginning of the pandemic, with 9.7 and 8.6 per 100,000 inhabitants, respectively. In addition to measures to cushion the economic consequences, regulations were also enacted that allowed hospitals a greater scope of action in workforce planning, such as the suspension of the Nursing Staff Lower Limit Ordinance (*Pflegepersonaluntergrenzen-Verordnung, PpUGV*) [9] and the relaxation of the Working Hours Act for certain sectors with the introduction of the COVID-19 Working Hours Ordinance (*COVID-19 Arbeitszeitverordnung, COVID-19-ArbZV*) [10].

As a result of the acute information demand on the hospitals’ management of COVID-19, the WHO Regional Office for Europe and the German Society of Hospital Disaster Response Planning and Crisis Management (*Deutsche Arbeitsgemeinschaft Krankenhaus Einsatzplanung, DAKEP*), among others, published comprehensive recommendations to support hospitals in the preparation and adjustment of emergency plans [11, 12]. Two recent cross-country comparisons of European countries, for example, show that all states used a variety of measures to create sufficient physical infrastructure and to increase workforce surge capacity at the beginning of the pandemic [13, 14]. All countries designated COVID-19 units and expanded hospital and surge capacities by setting-up additional acute and ICU beds within existing facilities [13]. In addition, Germany established a nationwide intensive care bed registry [15] and carried out inter-hospital transport of COVID-19 patients, including patient uptakes from abroad [13, 16]. With respect to the recruitment of additional staff, common strategies of countries were to augment the capacity of available health workers and the recruitment of medical and nursing students [14]. For Germany, redeployment of personnel who have already retired, initiatives for trained foreign personnel and calls for volunteers were also described. The cross-country comparisons mentioned here report individual measures with reference to country examples but do not provide an overall view of the countries in detail. Site-specific studies for Germany are rare thus far. The only published study in this context by Köppen et al. [17] analyzed pandemic preparedness planning and action at the federal and state level in Germany and found that measures to expand workforce capacity varied widely among the states. The analyses were based on data from websites of the German Federal and State Ministries for Health and of public health facilities. As with the cross-country comparisons, there is a lack of an overall overview here as well. In addition, we are not aware of any study that provides information on the extent to which measures described were actually applied and how their effectiveness was assessed.

In this study, we therefore systematically evaluated local concepts for expansion for intensive care bed capacity and staff recruitment during the first wave of the COVID-19 pandemic in Germany based on individual first-hand responses from hospitals themselves. The aims of our study were (1) to gain an overview of staffing in intensive care as well as the instruments/measures that were used to meet the increased staffing requirement during the first wave of the pandemic and (2) to assess how effective these strategies were perceived in practice, in order (3) to derive recommendations for future pandemics or crisis.

## Methods

The survey on recruitment was part of the nationwide survey study “ICU-Response”, which used a cross-sectional design and aimed to systematically assess local approaches to staff recruitment, training and safety management in the context of the first wave of the COVID-19 pandemic. This publication concentrates on the analysis of recruitment strategies; the results on training and safety management are reported elsewhere (e.g., [18]). ICU-Response was conducted as part of the national collaborative project egePan Unimed “Development, Testing and Implementation of regionally adaptive health care structures and processes for pandemic management guided by evidence and led by university clinics” in the University Medicine Network (*Netzwerk Universitätsmedizin, NUM*).

The study was approved by the Ethics Committee (EK 459/20) of the University Hospital RWTH Aachen.

### Sampling

The national intensive care register of the German Interdisciplinary Association for Intensive Care and Emergency Medicine (*Deutsche Interdisziplinäre Vereinigung für Intensiv- und Notfallmedizin, DIVI*; www.intensivregister.de) was used to identify all hospitals with intensive care capacities in Germany (> 1,200). Of those, a representative sample of 152 hospitals in total was formed. At first, an equal number of hospitals was selected from three regions (50 North, 48 Central, 54 South), each representing about one third of the nation’s population. While the North included the federal states of Lower Saxony, Schleswig-Holstein, Mecklenburg-Western Pomerania, Berlin, Hamburg, Bremen, Brandenburg, Saxony and Saxony-Anhalt, the central region comprised Thuringia, Hesse and North-Rhine Westphalia. The federal states of Bavaria, Baden-Wuerttemberg, Rhineland-Palatinate and Saarland belonged to the southern region.

Then, the hospitals’ level of care and COVID-19 burden were taken into consideration: Hospitals in Germany are assigned to different levels of care according to their specialization and the services they offer [19]. Depending on the federal state, three or four levels are distinguished. We attempted to map the proportions of hospital types and included 41 university hospitals or tertiary care hospitals, 40 secondary care hospitals, and 71 primary or basic care hospitals/hospitals with narrow specialization. To identify different levels of COVID-19 patient load, registry data from the Robert Koch Institute (RKI) for the period from January 1 to June 30, 2020, which were made available upon request, were used. Based on these, hospitals were grouped into hospitals with low (0–19), medium (20–59), or high (≥ 60) COVID-19 ICU patient burden. As hospitals with a high volume of COVID-19 patients (*N* = 52) were of particular interest, all of them were included in the survey without exception. The sample was supplemented by 49 hospitals with medium and 51 hospitals with low COVID-19 patient volumes.

### Online survey

The hospitals selected were invited to participate in the ICU-Response Survey via email with a personal letter in late March/early April 2021. They were asked to provide the name of a central contact person from their hospital’s critical care department for further correspondence. The central contacts were then contacted by email, provided with the questionnaire on recruitment for preview and asked to complete it online via *SoSci Survey* [20, 21]. Hospitals that had not responded after the initial invitation were reminded by email and/or contacted via telephone. Data collection was conducted between March 24 and June 20, 2021.

### Outcome parameters / study variables

The questionnaire was developed purely empirically by the authors based on their COVID-19/clinical experience, their experience in human resource management and in dialogue with further project collaborators. It was provided in German language and comprised a total of 24 questions on the intensive care bed situation (5 items), critical care staffing situation (6 items), recruitment strategies (6 items) and reallocation/shifting of personnel, that were closed-ended or semi-closed. The types of closed-ended questions used included trichotomous, single-choice and multiple-choice questions, as well as a Likert scaled question assessing recruitment methods. The structure of the questionnaire and outcome parameters are presented in Table 1; for the complete questionnaire (German original version and English translated version), please see Additional file 1.

**Table 1:** Outcome parameters assessed in the web-based questionnaire

Intensive care bed capacity	Items covered occurrence of bed closures due to staff shortages before the pandemic (Jan./Feb. 2020), activation of intensive care beds in existing ICUs considered as non-operational as of February 2020, preparation of new ICUs by upgrading recovery rooms and operating rooms as well as outside the hospital. Hospitals who answered the questions on preparing new ICUs with “Yes” were asked to provide more detailed information for each on number of beds prepared, maximum number of beds occupied and duration (in days) of bed occupancy.
Personnel situation in intensive care medicine	Hospitals were asked to provide information for different health care professional groups on the following items: total number of full-time positions (FTP) allocated to critical care in your hospital BEFORE the pandemic, number of vacancies in critical care medicine in your hospital BEFORE the pandemic, number of positions regularly filled with temporary workers in your hospital BEFORE the pandemic, additional staffing requirements (in FTP) that had arisen in your hospital in critical care medicine in the context of the pandemic, number of additional positions that were actually filled at your hospital during the pandemic. Furthermore, they were asked to indicate whether changes of the nurse-to-patient ratio (patients per nurse) or the nursing-skill-mix (ratio of specialists to assistants) of the staff in intensive care medicine had occurred in their hospital. Hospitals who answered with “Yes” were asked to specify this in more detail on the basis of type and scope as well as duration.
Staff recruitment and evaluation of recruitment measures	Instruments to cover the staffing needs during the pandemic, types of reallocation/shifting of personnel, strategies and instruments used in the context of recruiting new employees were assessed by using multiple-choice questions. Hospitals were asked to evaluate the effectiveness of recruitment measures in the context of the pandemic in a matrix format, in which several strategies were listed. Ratings were carried out on a 5-point-Likert scale, ranging from 1 (not effective at all) to 5 (very effective). Additionally, the answer categories “Type of recruitment did not take place” and “No information possible” were offered for selection for each item. Hospitals were also asked to report on the use of special incentives and whether it has been possible to attract new employees on a permanent basis as a result of the recruiting measures (by indicating the numbers for different health care professional groups).
Reallocation/shifting of personnel	In this section, the hospitals were asked to indicate which medical and nursing staff were shifted to intensive care. If the questions about staff from specific departments or with specific qualifications were answered with “Yes”, more detailed information was requested in each case about the maximum number of full-time positions and staff as well as the length of the assignment.

### Data analysis

Data analysis was performed using MS Excel 2019; figures were prepared with GraphPad Prism 5 (GraphPad Software, San Diego, CA, USA). Due to the exploratory nature of our study and research questions, we mainly performed descriptive data analysis. For hospitals that had called up the questionnaire several times, only the completed data set was included in the analysis. Data sets that had not been completed by the central contact persons were excluded. Questions that had been answered by less than 50% of participating clinics were also excluded from the analysis.

For reasons of comparison, the ratio of the number of full-time positions and the number of intensive care beds was calculated for each occupational group and hospital. To present the situation regarding personnel before the pandemic, the staffing data were related to the number of ICU beds set up (as of January 1, 2020); for the additional staffing requirements during the pandemic, the total number of currently operable ICU beds (low-care and high-care; as of April 24, 2020) served as reference value. The key data on the number of intensive care beds were derived from the DIVI Intensive Care Bed Registry and were made available by the RKI.

Data on full-time positions, number of employees and duration of deployment are given as medians, since this parameter better reflects the majority of hospitals in our data set compared to the mean.

## Results

Of a total of 152 hospitals requested, 41 participated in the survey (response rate 27.0%). One main question and 8 sub-questions (“if yes” questions) could not be evaluated since the response rates or number of data sets were too low.

An overview of the participating hospitals in terms of geographic location, level of care and Covid-19 patient volume is shown in Table 2.

**Table 2:** Basic characteristics of participating hospitals

	%; n/N
**Hospital location (region)**	
North	44%; 18/41
Middle	10%; 4/41
South	46%; 19/41
**Level of care** ^1^	
Primary care hospitals^2^ / hospitals with narrow specialization^5^	44%; 18/41
Secondary care hospitals^3^	17%; 7/41
University and tertiary care hospitals^4^	39%; 16/41
**Level of Covid-19 patient load (number of Covid-19 ICU patients)**	
Low (0–19)	39%; 16/41
Middle (20–59)	34%; 14/41
High (≥ 60)	27%; 11/41
**Professional affiliation central contact person**	
Physicians	95%; 39/41
Healthcare and nursing staff	2%; 1/41
Others	2%; 1/41

^1^ In Germany, general hospitals can be assigned to different levels of care according to their specialization and the services they offer. With variations between the federal states, the hospital laws regulate three or four levels of care [22, 23]:

^2^ The term summarizes basic and regular care hospitals. Hospitals for basic care ensure care in the fields of internal medicine and general surgery (corresponding in Germany to *Grundversorgung*). Hospitals providing standard/regular care must also operate other specialized departments, mostly for gynecology and obstetrics as well as otolaryngology, ophthalmology or orthopedics (*Regelversorgung*)

^3^ Secondary care hospitals cover an even broader spectrum, including specialist departments for pediatrics and neurology (*Schwerpunktversorgung*)

^4^ Tertiary care hospitals, such as university hospitals, have all specialties available and offer services for the treatment of rare or severe diseases as well (*Maximalversorgung*)

^5^ Hospitals with narrow specialization are specialized in certain fields of medicine (*Fachkliniken*), e.g. children’s hospitals and pulmonary clinics. They are not assigned to any level of care

### Intensive care bed capacity

**Fig. 1:**
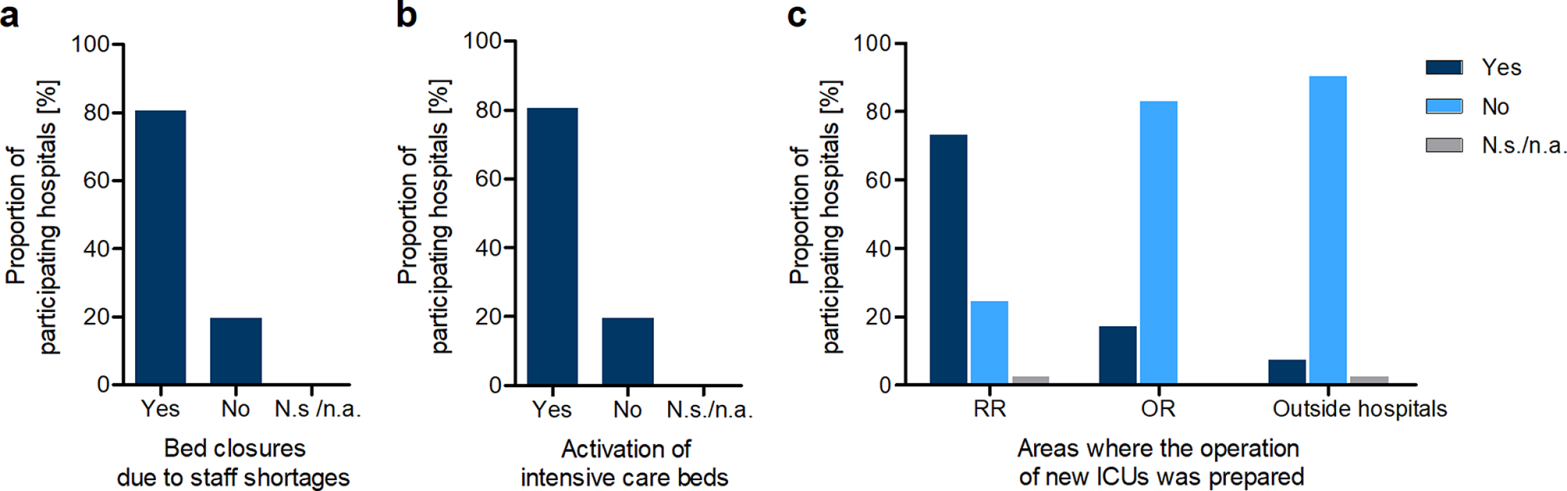
Measures to expand intensive care capacities during the first wave of the COVID-19 pandemic. (**a**) Hospitals were asked whether bed closures due to critical care staffing shortages occurred prior to the pandemic in their hospital (in January and February 2020). Answers are given in percentage terms. *n* = 41, N.s./n.a. Not specified/not answered. The median number of beds blocked per day was 4 (data not shown in the graph). (**b**) Hospitals were asked if intensive care beds had been activated in their hospital during the pandemic which were still considered inoperable as of February 2020. Answers are given in percentage terms. *n* = 41, N.s./n.a. Not specified/not answered. (**c**) Hospitals were asked whether they were preparing to run new ICUs in recovery rooms (RR), operating rooms (OR) and outside the hospital (e.g., in mess halls). Answers are shown as percentages for each area. *n* = 40–41, N.s./N.a. Not specified/not answered

Even before the pandemic (January and February 2020), bed closures due to staff shortages in intensive care occurred in 80.5% of the hospitals surveyed (see Fig. 1a). On median, four beds per day had to be closed.

The additional need for intensive care beds in the course of the pandemic was mainly met by activating intensive care beds previously (February 2020) considered as non-operational in existing ICUs (80.5% of the participating hospitals) and by upgrading recovery rooms (73.2% of the participating hospitals). A median of 8 intensive care beds were prepared in recovery rooms, but they were not occupied. Other measures, such as upgrading operating rooms (17.1%) and preparing external ICUs (7.3%), were taken by a comparatively lower proportion of the participating hospitals (see Fig. 1b-c). Due to their low occurrence, there are only a few data sets on the number of prepared ICU beds, occupancy rate and duration of occupancy, which do not allow any valid statements to be made.

### Personnel situation in intensive care medicine before and during the pandemic

**Fig. 2:**
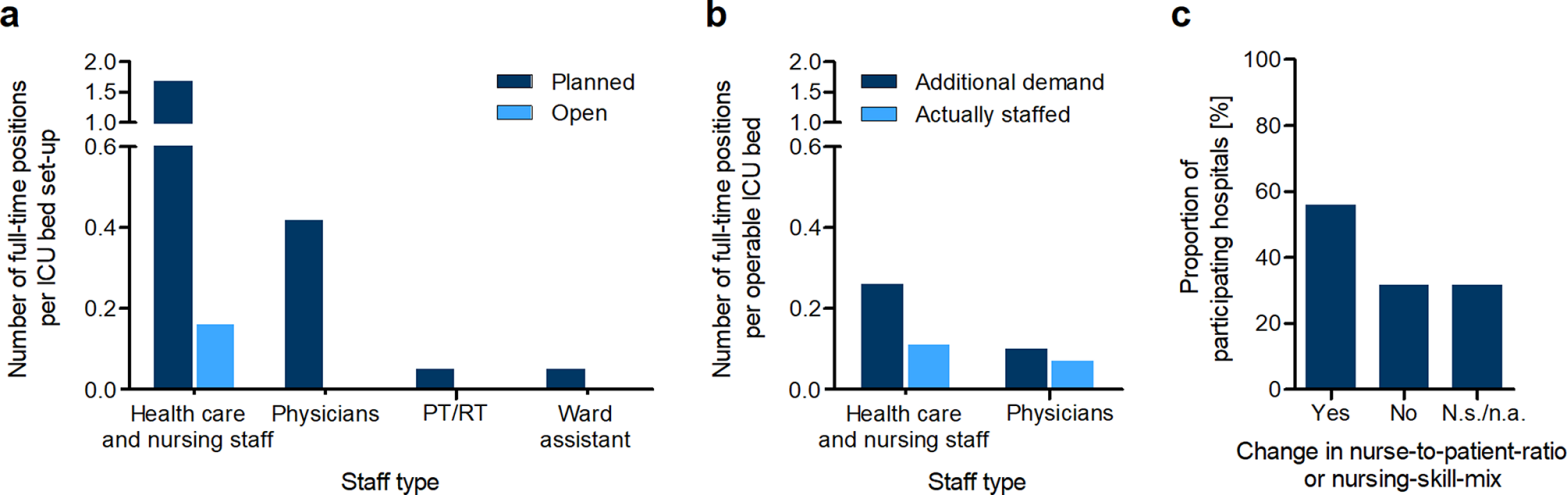
Staffing situation in critical care before and during the first wave of the COVID-19 pandemic. (**a**) Number of designated and vacant positions (full-time) for critical care medicine before the pandemic. Hospitals were asked to provide corresponding information on the specified professional groups in their hospital. The number of positions was related to the number of ICU beds set up (as of January 1, 2020); the medians of the hospitals are shown (*n* = 24–29). PT/RT Physiotherapists/respiratory therapists. (**b**) Additional staff requirements (full-time) in intensive care medicine during the pandemic and actual filling of the additional positions (through reallocation/shifting and through recruitment/new hires). The hospitals were asked to provide corresponding information on the specified occupational groups in their hospital. The number of positions was related to the total number of currently operable intensive care beds (as of April 24, 2020); the medians of the hospitals are shown (*n* = 26–29). No additional demand was identified for the staff groups of physiotherapists/respiratory therapists, ward assistants and others (data not shown). (**c**) The hospitals were asked whether the nurse-to-patient ratio or nursing-skill-mix of the staff in intensive care medicine had changed at their hospital. Answers are given in percentage terms. *n* = 39, N.s./n.a. Not specified/not answered

A median of 42 full-time positions for healthcare and nursing staff, 12 full-time positions for physicians, 1 full-time position for physiotherapists (PT)/respiratory therapists (RT) and 1.5 full-time positions for ward assistants were provided for intensive care per hospital before the pandemic (absolute numbers). After standardization, at median 1.68 full-time positions per ICU bed set up were planned for the occupational group of healthcare and nursing staff. Of these, 0.16 positions could not be filled (vacancy rate 9.5%). In the occupational group of physicians, a median of 0.4 full-time positions per ICU bed set up were included in the staffing plan, all of which could be filled (vacancy rate 0%). In the occupational groups of PT/RT and ward assistants, 0.05 full-time positions per ICU bed set up were planned, which were also filled at median (vacancy rate 0%) (see Fig. 2a).

When asked for temporary staff, approximately one third of the hospitals that provided information on this (9/29; 31.0%) had employed temporary staff primarily in the nursing service of their ICUs (median 9.3 full-time positions). Temporary staff was employed to a much lesser extent in the PT/RT group (median 3.0 full-time positions) and in the medical service (median 1 full-time position) (data not shown).

The increased demand for intensive care beds due to the pandemic also led to an increase in the demand for nursing and medical staff. In the occupational group of healthcare and nursing staff, a median demand of 0.26 additional full-time positions per operable ICU bed (as of April 24, 2020) had developed. Regarding the occupational group of physicians, a median demand of additional 0.10 full-time positions per operable ICU bed was found. While the majority of the additional positions required in the medical service (0.07 full-time positions per operable ICU bed) could be filled by reallocating/shifting staff and by recruiting/employing new staff, only 42% of the nursing staff (0.11 full-time positions per operable ICU bed) could be done so (see Fig. 2b). Additional demand for the staff groups of PT/RT and ward assistants could not be identified (data not shown).

In addition to increasing the number of personnel, changes of the staffing ratios were also used. Slightly more than half of the hospitals (56.1%) reported a change in the nurse-to-patient ratio or nursing-skill-mix (see Fig. 2c). Since further data on this are partly incomplete, no more precise statements can be made with regard to how and to what extent the ratio(s) had changed.

### Staff recruitment

**Fig. 3:**
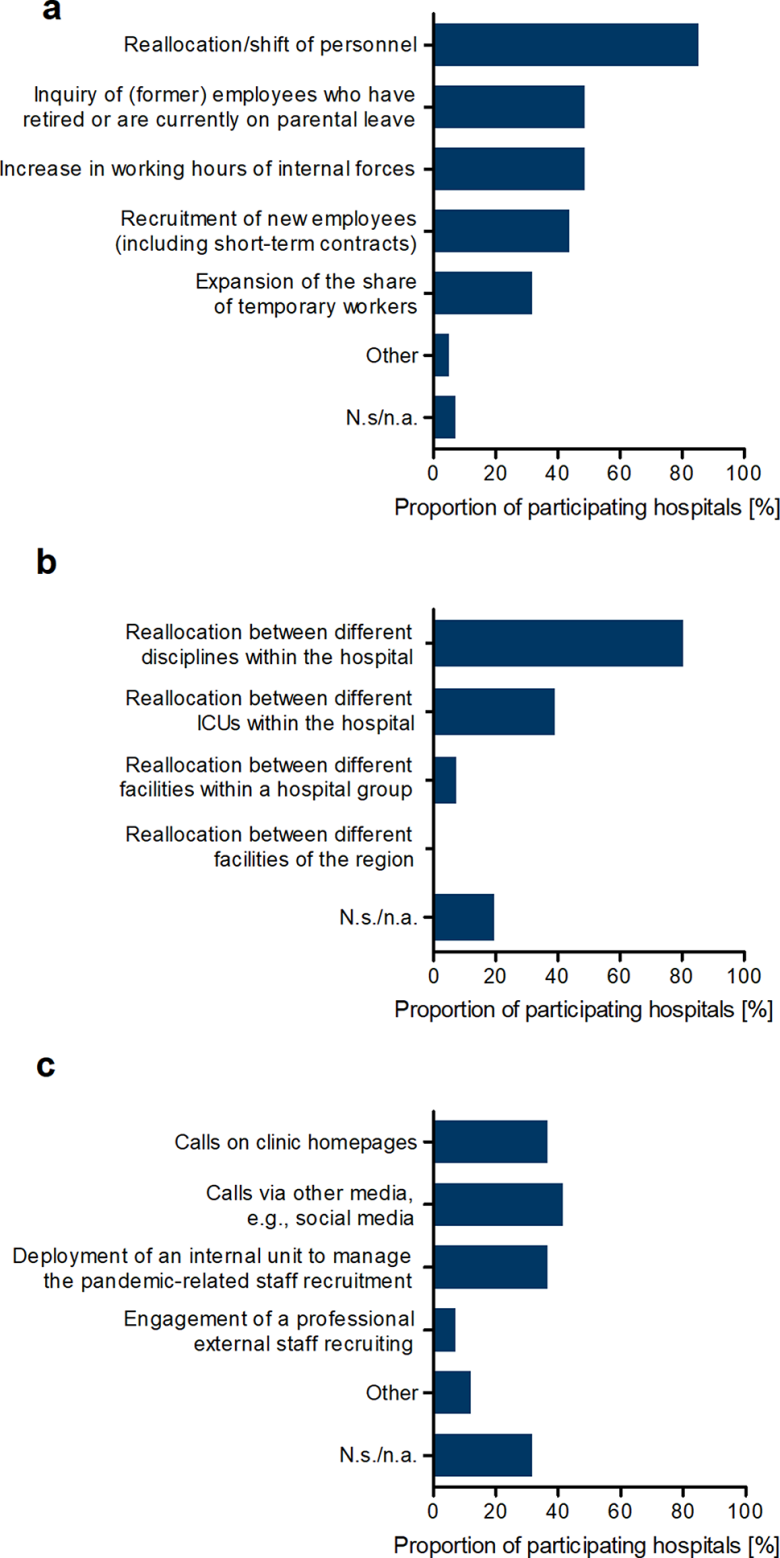
Recruitment efforts in the first wave of the COVID-19 pandemic. (**a**) Instruments and strategies used to meet staffing needs that were indicated by participating hospitals. Answers are given in percentage terms; multiple answers were possible. *n* = 38, N.s./n.a. Not specified/not answered. (**b**) Types of reallocation/shifts of medical personnel in the participating hospitals. Answers are given in percentage terms; multiple answers were possible. *n* = 35, N.s./n.a. Not specified/not answered, ICUs intensive care units. (**c**) Strategies and instruments used in the context of recruiting new employees that were reported by participating hospitals. Answers are given in percentage terms; multiple answers were possible. *n* = 39, N.s./n.a. Not specified/not answered

When asked what measures were used to meet staffing needs during the pandemic, reallocation/shifting of staff was cited most often (85.4%), followed by requesting former staff retired or currently on parental leave (48.8%), increasing the working hours of internal staff (48.8%) and new recruits (incl. temporary contracts; 43.9%). Temporary staffing increased in 31.7% of the participating hospitals (see Fig. 3a).

The reallocation/transfer of staff played a central role in covering the personnel requirements. Shifts of staff between different disciplines within a hospital occurred in 80.5% of participating hospitals. Moreover, 39% indicated that they had transferred staff between different ICUs within their hospital. The transfer of staff between different facilities within a group and between different facilities in a region took place only in a small proportion of hospitals (7.3%) and in none of them, respectively (see Fig. 3b).

Furthermore, hospitals were asked which strategies and instruments they used as part of the recruitment of new staff. In addition to calls on clinic homepages (36.6%) and in other media, e.g. social media (41.5%), 36.6% of the hospitals reported that they had established an internal office to manage pandemic-related staff recruitment. The involvement of an external personnel recruiter took place in only 7% of participating hospitals (see Fig. 3c).

Special incentives for staff recruitment were used by 17.1% of the hospitals (data not shown). Those came in form of cash benefits and/or additional leave.

### Reallocation/shift of personnel

**Fig. 4:**
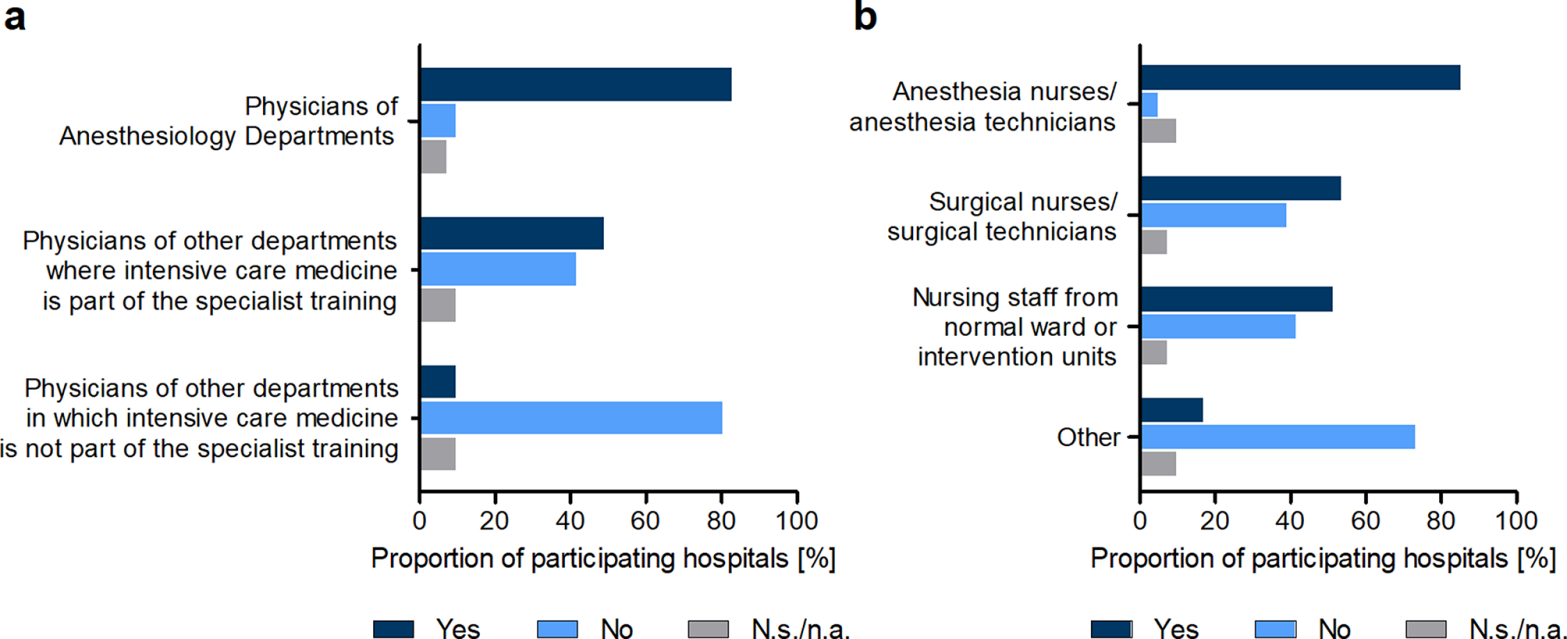
Reallocation of personnel to intensive care units during the first wave of the COVID-19 pandemic. (**a**) Reallocation of physicians according to their professional qualification. Hospitals were asked whether physicians of anesthesiology departments, physicians of other departments where intensive care medicine is part of the specialist training and physicians of other departments in which intensive care medicine is not part of the specialist training were deployed to ICUs in their hospital. Answers are given in percentage terms for each group. *n* = 39, N.s./n.a. Not specified/not answered. (**b**) Reallocation of nursing staff according to their professional qualification and of other employees. Analogously, the hospitals were asked whether anesthesia nurses and anesthesia technicians (corresponding in Germany to *Anästhesie-Fachpflegekräfte* bzw. *Anästhesie-Technische Assistenten (ATAs)*), surgical nurses and surgical technicians (in Germany *OP-Fachpflegekräfte* bzw. *Operationstechnische Assistenten (OTAs)*) as well as nursing personnel from normal wards or intervention units (e.g., cardiac catheterization lab) were deployed to ICUs in their hospital. Answers are given in percentage terms for each group. *n* = 39, N.s./n.a. Not specified/not answered

In 82.9% of hospitals, physicians from anesthesiology departments, who are normally assigned to the operating room, were appointed to the ICU (Fig. 4a). A median of five full-time positions or five anesthesiologists were deployed over a period of about 8 weeks. In about one in two hospitals (48.9%), also physicians from other disciplines, in which intensive care medicine is part of the specialist training, were transferred to the ICU. In this case, the number of data sets was too small to make a valid statement regarding the number and corresponding duration of deployment of the respective staff members.

The reallocation of doctors without intensive care training was negated by 80.5% of participating hospitals (see Fig. 4a).

Among nursing staff, particularly anesthesia nurses or anesthesia technicians were deployed in the ICU (85% of participating hospitals) (see Fig. 4b). A median of five full-time positions or six staff members at maximum were assigned over a period of 6 weeks.

In about every second hospital, surgical nurses or surgical technicians (53.7%) as well as nursing staff from normal wards or intervention units (51.2%) were assigned to intensive care medicine (see. Fig. 4b). Here again, the data sets were too small to make a valid statement regarding the number and corresponding duration of deployment of the respective staff members. The reallocation of other personnel was negated by 73.2% of participating hospitals (see Fig. 4b).

### Evaluation of recruitment measures

**Fig. 5:**
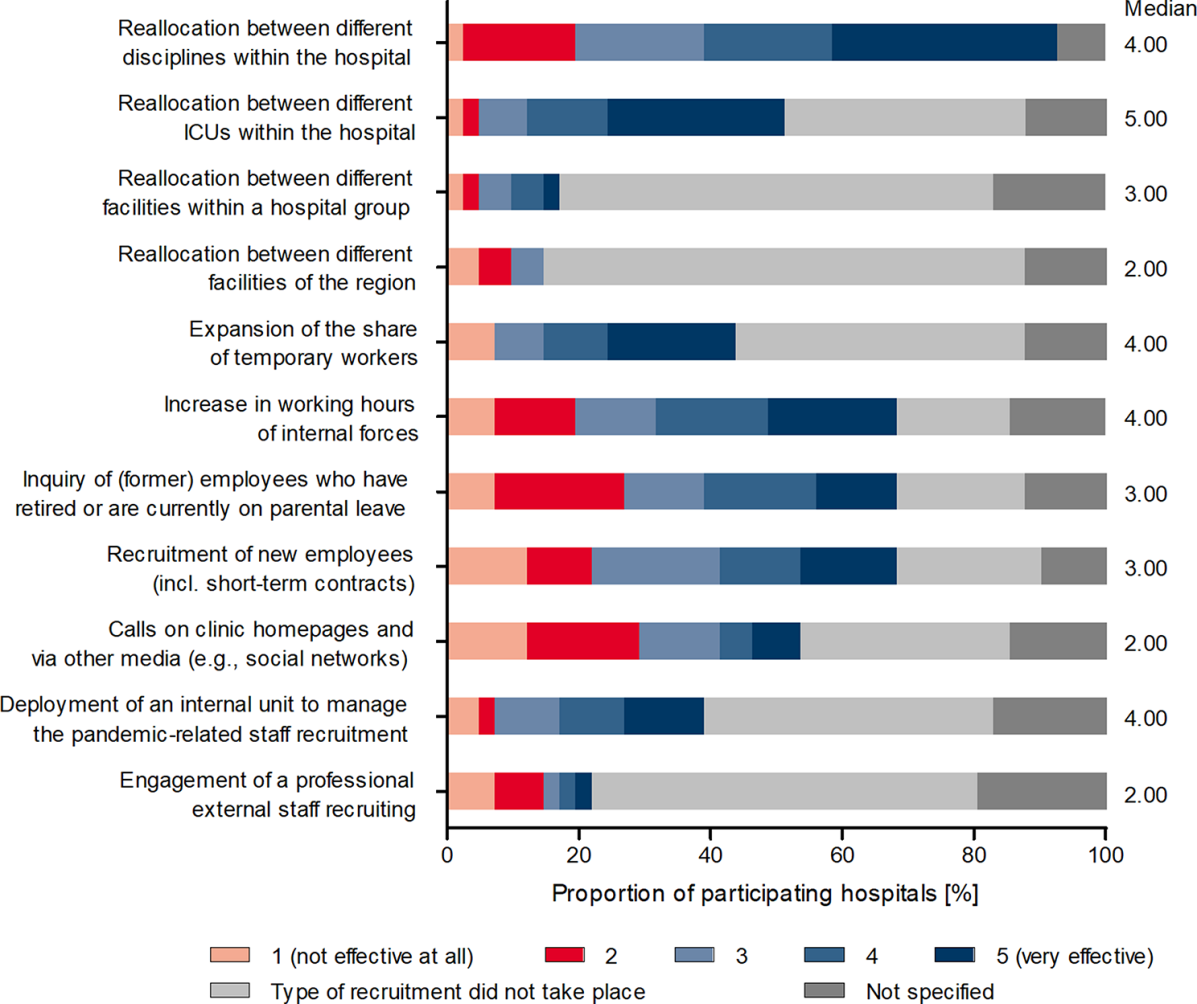
Assessing the effectiveness of recruitment interventions and strategies. Hospitals were asked to rate the effectiveness of various recruitment measures and strategies during the first wave of the pandemic using a scale of 1 (*not very effective at all*) to 5 (*very effective*). Data sets from 39 of the 41 participating hospitals were included in the analysis. Only those participating hospitals that provided a rating (from 1 to 5) were considered in determining the median

Reallocations/transfers between different disciplines within a hospital and between different ICUs emerged as the most effective measures in the pandemic (Fig. 5). On a scale from 1 to 5, with 1 being not effective at all and 5 being very effective, both instruments were rated 4 or 5 by 53.7% (shifting between different disciplines) and 39% (shifting between different ICUs) of the participating hospitals, whereby transfer of personnel between different disciplines took place in nearly every hospital. Expanding the use of temporary staff and increasing the hours of internal staff were each rated at 4 or 5 by approximately one third of the clinics, followed by inquiring of former staff retired or currently on parental leave (29.3%), recruiting/new staff (26.8%), and using an internal position to manage pandemic-related staff recruitment (22.0%). Conventional strategies such as appeals on homepages or through other media (including social media) were rated 1 or 2 by 29.3% of participating hospitals. In comparison, only 12.2% of the clinics rated these as 4 or 5.

Other measures such as reallocation between different facilities of a group or between different facilities of a region as well as the engagement of a professional external personnel recruiting were not used by the majority of the participating hospitals, the significance with regard to their efficiency is therefore very limited.

## Discussion

In this study, we assessed the staffing situation as well as local concepts for staff recruitment and their effectiveness throughout German ICUs during the first wave of the COVID-19 pandemic. The results from this nationwide analysis showed that the activation of ICU beds, which were previously considered non-operational (due to staff shortages), and the preparation of recovery rooms played a central role in expanding pandemic-related ICU bed capacity. In preparation of the expected rise in infections at the beginning of the pandemic, the federal and state governments had decided to double the number of intensive care beds by building up temporary intensive care capacity [24]. The preparation of recovery rooms to increase intensive care capacities is also listed among the recommendations on “Hospital Operational Planning and Crisis Management”, which was released by the Federal Office of Civil Protection and Disaster Assistance [11, 25]. Also, by international standards, upgrading recovery rooms appears to be a key element in rapidly increasing intensive care capacity in times of crisis [26–29].

According to our data, the construction of external ICUs and the preparation of operating rooms was, in comparison, of minor importance. Only 3 out of the 41 hospitals surveyed confirmed the operation/construction of external ICUs. These hospitals represent university or tertiary care hospitals that were classified as hospitals with middle (1) and high COVID-load (2) and/or hospitals that were located in highly affected regions during the first wave of the pandemic. Only one (high COVID-load) of these three hospitals actually used their external ICU for COVID patients.

From a global perspective, the conversion of facilities to external ICUs depends on regional infrastructure and politics. While for Israel, for example, the use of external ICUs was reported through repurposing existing infrastructure, such as an underground parking lot that was otherwise used as an emergency shelter hospital in times of war [30], this would not have been conceivable in Germany. Here, for example, exhibition halls and sports halls were rebuilt in COVID-19 centers [31]. The preparation of operating rooms was a key element in enhancing ICU capacity in other countries as well. Lefrant et al. reported that 32% of new ICU beds were created by upgrading operating rooms in France [29]. Likewise, in Italy, Carenzo et al. [28] described that ICU capacity had been increased substantially by converting operating rooms.

The extension of intensive care capacity is accompanied by an increasing need for medical and nursing staff. The results of the present study show that in particular, the shortage of nursing staff, which was already present before the pandemic, exacerbated during the pandemic, despite various recruitment efforts. A similar picture emerges in other countries, e.g. Australia [32]. Despite the activation of ICU beds, the quality of intensive care treatment in many places does not meet the pre-pandemic standards. Impairments in the quality of care are due to staff shortages, high workloads, inadequate provision of protective equipment for staff, shortage of medication and ventilation equipment, as well as knowledge deficits due to the novelty of the disease and lack of experience/routines [33, 34]. Nursing staff reported improvised conditions, situations that put patients at risk and the fear of making mistakes [34]. The enormous workload, the new and challenging working conditions and the fear of infecting oneself and loved ones such as family and friends have also led to an increased susceptibility of healthcare professionals to psychological stress, which has resulted in higher prevalence rates for anxiety, depression, burn-out, acute stress disorder and post-traumatic stress disorder [35]. According to a study by Lai et al. [36], nursing staff, women and front-line workers have a higher risk of developing psychological stress, presumably due to more intensive patient contact, a higher risk of infection and fewer opportunities for codetermination. Women are also disproportionately represented in the nursing profession. The enhanced wearing of protective equipment is of particular importance, as it is also experienced as physically very stressful [34] and described in connection with communication difficulties, a negative impact on personal performance and on physical health (e.g., exhaustion, headache, breathlessness) [37]. In the subsequent COVID waves, persistent psychological stress was identified [35, 38]. In addition to efforts to recruit additional staff, efforts to relieve the burden on nursing staff should therefore include offers of peer psychosocial support, the implementation of team concepts to strengthen cohesion, resilience and appreciation, as well as other measures.

To cover the increased personnel requirements in the short-term, various measures and strategies were taken by German hospitals. The reallocation/shifting of personnel, primarily between different disciplines within hospitals, played a central role and was used in almost all of the participating hospitals in our survey. Further measures that were implemented, albeit less frequently, included inquiring of former employees who had already retired or were currently on parental leave, extending the working hours of internal forces, recruiting new staff (incl. short-term contracts) and expanding the share of temporary employment. For the implementation of some measures, a change or suspension of the existing legislation was necessary. The change in the nurse-to-patient ratio or the nursing-skill-mix, for example, was enabled by a temporary suspension of the PpUGV [9] and took place in every second of the participating hospitals. The aim was to enable hospitals to adjust their workflows at very short notice and to briefly relieve them of the requirements for nursing staff deployment in care-sensitive areas. Effective August 1, 2020, the regulations for critical care and geriatrics were reinstated to avoid understaffing in nursing and jeopardizing the particularly vulnerable patients to be treated in these two areas. The nurse-to-patient ratio defines the maximum number of patients per nurse, while the nursing-skill-mix represents the ratio of nursing and auxiliary staff. Until January 31, 2021, the PpUGV provided a maximum of 2.5 patients per nurse during day shifts or 3.5 patients per nurse during night shifts in intensive care [9].

The measures reported here are in line with previous reports using information from websites of Federal and State Ministries of Health and public health facilities or data from the COVID-19 Health System and Response Monitor platform [13, 14, 17]. These studies also list further strategies for Germany, such as the recruitment of trained foreign personnel and support by medical personnel from the military, which, however, have not been explicitly asked for in our study. Countries in the European region and Canada adopted at least two or more measures in combination [14].

With regard to the reallocation of staff, personnel from anesthesiology departments (physicians and nursing staff) have been primarily deployed in ICUs. This is mainly due to the fact that intensive care is a prominent part of training for both anesthesiologists as well as anesthesia nurses and that at the same time elective surgeries and interventions had been cancelled at a very early stage of the pandemic in Germany (with a low number of COVID patients), leading to a freeing of personnel resources. Additionally, medical staff trained in intensive care medicine from other specialties as well as surgical nurses/surgical technicians and nurses from general wards and intervention units were temporarily shifted to ICUs in one out of two participating hospitals. Reallocation, however, is not entirely unproblematic, as patients and tasks in ICUs require specific expertise, incl. handling of a variety of medical equipment, and experiences. Takeover of tasks by non-specialist nurses and physicians should therefore only be applied with appropriate training in intensive care. In Germany, working on an ICU formally requires a completed 3-year vocational training as a general nurse [39] or, equivalently, a Bachelor’s degree in nursing/nursing science. This can be supplemented by a 2-year specialist further training in “Intensive Care” or “Intensive and Anesthesia Care”. According to legal regulations [40, 41] and recommendations by the DIVI [42], a certain minimum proportion of nurses with additional specialist further training must be available in the nursing team in ICUs on each shift.

While previous studies on expanding and securing staff capacity mainly concentrated on qualitative analyses of the measures used, our study also evaluated their efficiency in practice based on individual ratings of participating hospitals. The findings on this reflect subjective perceptions which are presumably geared more towards the professional group of physicians as 95% of the central contacts were doctors in management positions in the field of intensive care medicine (see Table 2). Our results uncover that the reallocation of staff between different disciplines within a hospital and between different ICUs within a hospital were rated as the most effective recruiting measures, whereby reallocation between different disciplines was used by the majority of participating hospitals. Compared to this, reallocation of staff between different ICUs occurred somewhat less, probably indicating some specialization of ICUs in the care for COVID patients. However, when comparing those findings with the data in Fig. 2b providing a more objective assessment, it becomes apparent that the reallocation alone is not sufficient to cover the additional demand for personnel. This is particularly visible in the nursing staff group.

Measures like “expansion of temporary employment”, “increase working hours of internal staff”, “inquiry with former employees who have retired or are currently on parental leave” and “new recruitments”, however, were perceived as less effective; reasons could be administrative efforts which are linked to these but also limited availability of former and new personnel as well as limited capacities for increasing working hours. Initiatives such as appeals on websites and social media were rated least effective. Target persons may not have felt personally addressed or have not used these communication tools.

Although the establishment of an internal position to manage the pandemic-related staff recruitment was reported by over a third of the respondents, it was comparably rated as ineffective by the majority. Maybe operating and communication processes need to be optimized to make this measure more efficient and provide stronger support in recruitment and training matters for hospitals in crisis situations. The New York City (NYC) Health and Hospitals organization, for example, which operates NYC’s public hospitals, has been very successful in implementing this tool with others as part of redesigned recruitment, onboarding, and training processes, and has been able to acquire a large number of additional staffing members [43].

When evaluating the recruitment strategy in this study, it should be noted that it only considers the increase in personnel, but does not take into account the quality of intensive care. Future studies should include this point (e.g., by recording quality indicators) and involve it in the overall assessment, as this is the only way to make statements about the actual effectiveness.

Special incentives (monetary and non-monetary) did not play a role in recruiting staff for the majority of respondents. Only a small proportion of respondents affirmed the use. How much additional staff could be recruited through this or whether the hospitals that used this measure were able to generate more staff is not answered by this study.

### Limitations

The response rate to the survey is only 27%, despite sending reminders to the central contact persons. This fact limits the representativeness of the results. In addition, the online questionnaire was sometimes answered incompletely. As a consequence, some questions could not be evaluated due to only few available data sets. One reason for non-participation or non-response to individual questions might have been the challenge to report detailed numeric data with regard to the staffing situation and intensive care bed capacity, which might have required some internal inquiry. Additionally, the COVID-19-related tense situation in the hospitals at the time of the survey might have prevented participation in individual cases.

Furthermore, the low response rate did not allow us to run subgroup analyses and further differentiate the results between the level of care of the hospitals and the corresponding COVID burden. The selection of an initial larger sample and/or a modified implementation strategy might be useful tools for future online surveys.

Other limitations are the transferability of the results and the lack of testing validity and reliability on the questionnaire. The results should primarily be considered in the context of hospitals in Germany. Generalization or transferability to other healthcare systems is limited due to the existing differences between the healthcare systems, including different training concepts for medical and nursing staff. The choice of closed and semi-open item response options as well as precisely formulated and clearly defined questions for collecting information were intended to ensure valid and reliable data, although statistical tests on these quality criteria were not carried out in advance of the survey.

Nevertheless, compared to previous reports, the data of our study were collected directly from the hospitals themselves and thus provide not only a qualitative but also a quantitative insight into the strategies and measures used for workforce planning during the first wave of the COVID-19 pandemic in Germany. In addition, the study provides, to our knowledge, for the first time an evaluation of which of the measures were perceived effective in practice. Of equal interest would have been the recruitment processes/measures used in subsequent waves of the pandemic and resulting changes, which may be the subject of future studies.

## Conclusions

The results of our study provide detailed insights into how hospitals in Germany managed the first Covid-19 wave with regard to the bed and staffing situation. By activating intensive care beds that previously considered inoperable due to staff shortages and preparing recovery rooms additional intensive care capacity has been made available. Furthermore, our findings reveal that the pandemic has exacerbated the existing shortage in nursing staff despite numerous recruitment efforts. This fact reflects a key issue that was and continues to be critical also in other settings.

Reallocation/shifting of staff within hospitals was a pivotal element in meeting staffing needs, although further measures are required in addition. Number and type of those employed may depend on several factors (e.g., local, structural and/or financial). Our findings provide an important and valuable decision-making aid to support healthcare providers and policymakers in preparing for and responding to future crises involving acutely increasing patient numbers and the need for rapid expansion of intensive care capacity.

### Electronic supplementary material

Below is the link to the electronic supplementary material.

Supplementary Material 1

## Data Availability

The data sets generated and analyzed during the current study are not publicly available due to reasons of data protection but are available from the corresponding author, CN, on reasonable request. Acceptance is subject to approval from participating hospitals.

## Abbreviations

DIVI: *Deutsche Interdisziplinäre Vereinigung für Intensiv- und Notfallmedizin*, German Interdisciplinary Association for Intensive Care and Emergency Medicine
FTP: Full-Time Position
ICU(s): Intensive Care Unit(s)
PpUGV: *Pflegepersonaluntergrenzenverordnung*, Nursing Staff Lower Limit Ordinance
PT: Physiotherapist
RKI: Robert Koch Institute
RT: Respiratory Therapist
WHO: World Health Organization
