# Supplementary material for: Workforce strategies during the first wave of the COVID-19 pandemic: a retrospective online survey at intensive care units in Germany
Source: BMC Health Serv Res. 2024 Apr 1;24:407. doi: 10.1186/s12913-024-10848-w (PMC10985885; doi:10.1186/s12913-024-10848-w)
Supplement: Supplementary file 1 — Supplementary Material 1 [file 12913_2024_10848_MOESM1_ESM.docx]

**Additional file 1**

**Appendix table 1** Questionnaire “Recruitment“

|  | **No.** | **Questions (German original version)** | **Questions (English translated version for publication purposes only)** |
| --- | --- | --- | --- |
| ICU bed situation | 1 | Kam es an Ihrem Krankenhaus VOR der Pandemie zu Bettensperrungen wegen Personalmangels in der Intensivmedizin (Jan. + Feb. 2020)?  ⭘ Ja – mit Zusatzfrage: Wie viele Betten mussten durchschnittlich pro Tag wegen Personalmangels gesperrt werden?  ⭘ Nein  ⭘ Keine Angaben möglich | Were there bed closures at your hospital BEFORE the pandemic due to critical care staffing shortages (Jan + Feb 2020)?  ⭘ Yes – with additional question: How many beds per day had to be closed on average due to staff shortages?  ⭘ No  ⭘ Not specified |
|  | 2 | Wurden an Ihrem Krankenhaus in der Pandemie (März-Juni 2020) auf bestehenden Intensivstationen Intensivbetten „aktiviert“, die noch im Februar 2020 als nicht betreibbar galten?  ⭘ Ja  ⭘ Nein  ⭘ Keine Angaben möglich | Were ICU beds "activated" at your hospital during the pandemic (March-June 2020) in existing ICUs that were not considered operable as recently as February 2020?  ⭘ Yes  ⭘ No  ⭘ Not specified |
|  | 3 | Wurde an Ihrem Krankenhaus der Betrieb neuer Intensivstationen vorbereitet, indem Aufwachräume aufgerüstet wurden?  ⭘ Ja  ⭘ Nein  ⭘ Keine Angaben möglich | Has your hospital prepared for the operation of new intensive care units by upgrading recovery rooms?  ⭘ Yes  ⭘ No  ⭘ Not specified |
|  | 3a | Falls ja: Zusätzliche Intensivbetten in Aufwachräumen  ⭘ Für wie viele Betten wurde hier vorbereitet? ___  ⭘ Wie viele davon wurden hier maximal belegt? (Wenn nur in Standby: bitte 0 („Null“) eintragen) ___  ⭘ Wie viele Tage waren hier Betten belegt? ___  ⭘ Keine Angaben möglich | If yes: Additional ICU beds in recovery rooms  ⭘ How many beds were prepared for here? ___  ⭘ What was the maximum number of beds occupied here? (If only in standby, please enter 0 („Zero“)) ___  ⭘ How many days beds were occupied here? ___  ⭘ Not specified |
|  | 4 | Wurde an Ihrem Krankenhaus der Betrieb neuer Intensivstationen vorbereitet, indem OP-Säle aufgerüstet wurden?  ⭘ Ja  ⭘ Nein  ⭘ Keine Angaben möglich | Has your hospital prepared for the operation of new intensive care units by upgrading operating rooms?  ⭘ Yes  ⭘ No  ⭘ Not specified |
|  | 4a | Falls ja: Zusätzliche Intensivbetten in OP-Sälen  ⭘ Für wie viele Betten wurde hier vorbereitet? ___  ⭘ Wie viele davon wurden hier maximal belegt? (Wenn nur in Standby: bitte 0 („Null“) eintragen) ___  ⭘ Wie viele Tage waren hier Betten belegt? ___  ⭘ Keine Angaben möglich | If yes: Additional ICU beds in operating rooms  ⭘ How many beds were prepared for here? ___  ⭘ What was the maximum number of beds occupied here? (If only in standby, please enter 0 („Zero“)) ___  ⭘ How many days beds were occupied here? ___  ⭘ Not specified |
|  | 5 | Wurde an Ihrem Krankenhaus der Betrieb neuer Intensivstationen außerhalb des Krankenhauses (z. B. Messehallen) vorbereitet?  ⭘ Ja  ⭘ Nein  ⭘ Keine Angaben möglich | Has your hospital prepared for the operation of new intensive care units outside the hospital (e.g., exhibition halls)?  ⭘ Yes  ⭘ No  ⭘ Not specified |
|  | 5a | Falls ja: Zusätzliche Intensivbetten außerhalb des Krankenhauses  ⭘ Für wie viele Betten wurde hier vorbereitet? ___  ⭘ Wie viele davon wurden hier maximal belegt? (Wenn nur in Standby: bitte 0 („Null“) eintragen) ___  ⭘ Wie viele Tage waren hier Betten belegt? ___  ⭘ Keine Angaben möglich | If yes: Additional ICU beds outside the hospital  ⭘ How many beds were prepared for here? ___  ⭘ What was the maximum number of beds occupied here? (If only in standby, please enter 0 („Zero“)) ___  ⭘ How many days beds were occupied here? ___  ⭘ Not specified |
| ICU staffing situation | 6 | Wie viele Vollkraftstellen (VK) waren in Ihrem Krankenhaus VOR der Pandemie für die Intensivmedizin insgesamt vorgesehen?  ⭘ Gesundheits- und Krankenpfleger*innen: ___  ⭘ Ärzt*innen: ___  ⭘ Physiotherapeut*innen/ Atmungstherapeut*innen: ___  ⭘ Stationsassistent*innen: ___  ⭘ Keine Angaben möglich | What was the total number of full-time positions (FTP) allocated to critical care in your hospital BEFORE the pandemic?  ⭘ Health care and nursing staff: ___  ⭘ Physicians: ___  ⭘ Physiotherapists/ respiratory therapists: ___  ⭘ Ward assistants: ___  ⭘ Not specified |
|  | 7 | Wie viele offene Stellen (in VK) gab es in Ihrem Krankenhaus in der Intensivmedizin VOR der Pandemie?  ⭘ Gesundheits- und Krankenpfleger*innen: ___  ⭘ Ärzt*innen: ___  ⭘ Physiotherapeut*innen/ Atmungstherapeut*innen: ___  ⭘ Stationsassistent*innen: ___  ⭘ Keine Angaben möglich | How many vacancies (in FTP) were there in critical care medicine in your hospital BEFORE the pandemic?  ⭘ Health care and nursing staff: ___  ⭘ Physicians: ___  ⭘ Physiotherapists/ respiratory therapists: ___  ⭘ Ward assistants: ___  ⭘ Not specified |
|  | 8 | Wie viele Stellen (in VK) waren in Ihrem Krankenhaus in der Intensivmedizin VOR der Pandemie regelmäßig mit Zeitarbeitskräften besetzt?  ⭘ Gesundheits- und Krankenpfleger*innen: ___  ⭘ Ärzt*innen: ___  ⭘ Physiotherapeut*innen/ Atmungstherapeut*innen: ___  ⭘ Stationsassistent*innen: ___  ⭘ Keine Angaben möglich | How many positions in critical care have been regularly filled with temporary workers in your hospital BEFORE the pandemic?  ⭘ Health care and nursing staff: ___  ⭘ Physicians: ___  ⭘ Physiotherapists/ respiratory therapists: ___  ⭘ Ward assistants: ___  ⭘ Not specified |
|  | 9 | Welcher zusätzliche Personalbedarf (in VK) hat sich in Ihrem Krankenhaus in der Intensivmedizin im Rahmen der Pandemie ergeben?  ⭘ Gesundheits- und Krankenpfleger*innen: ___  ⭘ Ärzt*innen: ___  ⭘ Physiotherapeut*innen/ Atmungstherapeut*innen: ___  ⭘ Stationsassistent*innen: ___  ⭘ Sonstige: ___  ⭘ Keine Angaben möglich | What additional staffing requirements (in FTP) have arisen in your hospital in critical care medicine in the context of the pandemic?  ⭘ Health care and nursing staff: ___  ⭘ Physicians: ___  ⭘ Physiotherapists/ respiratory therapists: ___  ⭘ Ward assistants: ___  ⭘ Other: ___  ⭘ Not specified |
|  | 10 | Wie viele zusätzliche Stellen (in VK) in der Intensivmedizin konnten in Ihrem Krankenhaus im Rahmen der Pandemie tatsächlich besetzt werden (durch Re-Allokation/Verschiebung UND durch Neurekrutierung/Neuanstellung)?  ⭘ Gesundheits- und Krankenpfleger*innen: ___  ⭘ Ärzt*innen: ___  ⭘ Physiotherapeut*innen/ Atmungstherapeut*innen: ___  ⭘ Stationsassistent*innen: ___  ⭘ Sonstige: ___  ⭘ Keine Angaben möglich | How many additional positions in intensive care medicine were actually filled at your hospital during the pandemic (through reallocation/shifting and through recruiting/new hires)?  ⭘ Health care and nursing staff: ___  ⭘ Physicians: ___  ⭘ Physiotherapists/ respiratory therapists: ___  ⭘ Ward assistants: ___  ⭘ Other: ___  ⭘ Not specified |
|  | 11 | Wurde an Ihrem Krankenhaus in der Intensivmedizin der „Pflegeschlüssel“ (Patienten pro Pflegekraft) oder der „Qualifikationsmix“ (Verhältnis Fach- zu Assistenzkräften) des Personals verändert?  ⭘ Ja  ⭘ Nein  ⭘ Keine Angabe möglich | Has the nurse-to-patient-ratio (patients per nurse) or the nursing-skill-mix (ratio of specialists to assistants) been changed at your hospital in intensive care?  ⭘ Yes  ⭘ No  ⭘ Not specified |
|  | 11a | Falls ja: Veränderung „Pflegeschlüssel“ (Patienten pro Pflegekraft) oder „Qualifikationsmix“ (Verhältnis Fach- zu Assistenzkräften)  ⭘ In welcher Weise und in welchem Umfang?  ⭘ Wie lange? (Angaben in Tagen)  ⭘ Keine Angaben möglich | If yes: Change in nurse-to-patient-ratio (patients per nurse) or nursing-skill-mix (ratio of specialists to assistants)  ⭘ In which way and to which extent?  ⭘ How long? (Data in days)  ⭘ Not specified |
| Recruiting | 12 | Welche Instrumente haben Sie zur Deckung der Personalbedarfe im Rahmen der Pandemie verwendet?  ⭘ Ausweitung des Anteils von Zeitarbeit  ⭘ Neurekrutierung/Neuanstellungen (inkl. Kurzzeitverträge)  ⭘ Aufstockung der Arbeitszeiten interner Kräfte  ⭘ Anfrage bei (ehemaligen) Mitarbeitern im Ruhestand oder aktuell in Elternzeit  ⭘ Re-Allokation/Verschiebung von Personal  ⭘ Sonstige: ___  ⭘ Keine Angabe möglich | What instruments have been used to meet staffing needs during the pandemic?  ⭘ Increase in working hours of internal forces  ⭘ Recruitment of new employees (incl. short-term contracts)  ⭘ Expansion of the share of temporary workers  ⭘ Inquiry of (former) employees who have retired or are currently on parental leave  ⭘ Reallocation/shifting of personnel  ⭘ Other: ___  ⭘ Not specified |
|  | 13 | Re-Allokation/Verschiebung von Personal  ⭘ Re-Allokation zwischen verschiedenen Fachdisziplinen innerhalb des Krankenhauses  ⭘ Re-Allokation zwischen verschiedenen Intensivstationen innerhalb des Krankenhauses  ⭘ Re-Allokation zwischen verschiedenen Einrichtungen innerhalb eines Konzerns  ⭘ Re-Allokation zwischen verschiedenen Einrichtungen der Region  ⭘ Keine Angaben möglich | Reallocation/shifting of personnel  ⭘ Reallocation between different disciplines within the hospital  ⭘ Reallocation between different intensive care units within the hospital  ⭘ Reallocation between different facilities within a hospital group  ⭘ Reallocation between different facilities of the region  ⭘ Not specified |
|  | 14 | Welche Strategie und welche Instrumente kamen im Rahmen der Neurekrutierung zum Einsatz?  ⭘ Aufruf auf der Homepage  ⭘ Aufruf über sonstige Medien (u. a. Social Media)  ⭘ Einsetzung einer internen Stelle zur Bewältigung des Pandemie-bedingten Personal-Recruitments  ⭘ Engagement eines professionellen externen Personal-Recruitments  ⭘ Sonstige: ___  ⭘ Keine Angaben möglich | What strategies and instruments have been used in the context of recruiting new employees?  ⭘ Calls on homepages  ⭘ Calls via other media (e.g., social media)  ⭘ Deployment of an internal unit to manage the pandemic-related staff recruitment  ⭘ Engagement of a professional external staff recruiting  ⭘ Other: ___  ⭘ Not specified |
|  | 15 | Wie wirksam schätzen Sie folgende Methoden im Rahmen der Rekrutierung von Personal im Rahmen von Pandemien ein?  [Skala von 1: überhaupt nicht wirksam bis 5: sehr wirksam], zusätzlich standen jeweils die Felder „Art der Rekrutierung fand nicht statt“ und „Keine Angaben möglich“ zur Auswahl  - Re-Allokation zwischen verschiedenen Fachdisziplinen innerhalb des Krankenhauses  - Re-Allokation zwischen verschiedenen Intensivstationen  - Re-Allokation zwischen verschiedenen Einrichtungen innerhalb eines Konzerns  - Re-Allokation zwischen verschiedenen Einrichtungen der Region  - Ausweitung des Anteils von Zeitarbeit  - Aufstockung der Arbeitszeiten interner Kräfte  - Anfrage bei (ehemaligen) Mitarbeitern im Ruhestand oder aktuell in Elternzeit  - Neurekrutierung/Neuanstellungen (inkl. Kurzzeitverträge)  - Aufruf auf der Homepage/Aufruf über sonstige Medien (u. a. Social Media)  - Einsetzung einer internen Stelle zur Bewältigung des Pandemie-bedingten Personal-Recruitments  - Engagement eines professionellen externen Personal-Recruitments | How effective do you consider the following methods to be in recruiting personnel in the context of pandemics?  [scale from 1: not effective at all to 5: very effective], additionally, for each item the fields  “Type of recruitment did not take place” and “Not specified” were available for selection  - Reallocation between different disciplines within the hospital  - Reallocation between different intensive care units  - Reallocation between different facilities within a hospital group  - Reallocation between different facilities of a region  - Expansion of the share of temporary workers  - Increase in working hours of internal forces  - Inquiry of (former) employees who have retired or are currently on parental leave  - Recruitment of new employees (incl. short-term contracts)  - Calls on homepages or via other media (e.g. social media)  - Deployment of an internal unit to manage the pandemic-related staff recruitment  - Engagement of a professional external staff recruiting |
|  | 16 | Haben Sie besondere Anreize (Incentives) zur Rekrutierung von Personal eingesetzt?  ⭘ Ja – Wenn ja, welche? ___  ⭘ Nein  ⭘ Keine Angaben möglich | Did you use special incentives to recruit staff?  ⭘ Yes – If yes, which ones? ___  ⭘ No  ⭘ Not specified |
|  | 17 | Konnten Sie durch die Rekrutierungsmaßnahmen dauerhaft neue Mitarbeiter für Ihr Klinikum gewinnen?  ⭘ Gesundheits- und Krankenpfleger*innen: ___ (Wie viele VKs?)  ⭘ Ärzt*innen: ___ (Wie viele VKs?)  ⭘ Physiotherapeut*innen/ Atmungstherapeut*innen: ___ (Wie viele VKs?)  ⭘ Stationsassistent*innen: ___ (Wie viele VKs?)  ⭘ Sonstige (z. B. Studierende, freiwillige Helfer*innen: ___ (Wie viele VKs?)  ⭘ Keine Angaben möglich | Were you able to attract new employees to your hospital on a permanent basis as a result of the recruiting measures?  ⭘ Health care and nursing staff: ___ (How many FTPs?)  ⭘ Physicians: ___ (How many FTPs?)  ⭘ Physiotherapists/ respiratory therapists: ___ (How many FTPs?)  ⭘ Ward assistants: ___ (How many FTPs?)  ⭘ Other (e.g., students, volunteers): ___ (How many FTPs?)  ⭘ Not specified |
|  |  | Die folgenden Fragen beziehen sich auf die Re-Allokation/Verschiebung von Personal | The following questions relate to the reallocation/shifting of personnel |
|  | 18 | Wurden an Ihrem Krankenhaus Ärzt*innen aus der operativen Anästhesie in die Intensivmedizin verschoben?  ⭘ Ja  ⭘ Nein  ⭘ Keine Angaben möglich | Have any physicians at your hospital been moved from operative anesthesia to critical care?  ⭘ Yes  ⭘ No  ⭘ Not specified |
|  | 18a | Falls ja: Re-Allokation von Anästhesist*innen aus der operativen Versorgung auf die Intensivstation  *Wenn keine genauen Zahlen vorhanden sind, reichen Schätzungen.*  ⭘ Wie viele VK maximal? ___  ⭘ Wie viele Mitarbeiter*innen maximal? ___  ⭘ Wie lange? (in Wochen) ___  ⭘ Keine Angaben möglich | If yes: Reallocation of anesthetists from surgical care to the intensive care unit  *If no detailed data are available, please estimate.*  ⭘ How many FTP maximum?  ⭘ How many employees maximum? ___  ⭘ How long? (in weeks) ___  ⭘ Not specified |
|  | 19 | Wurden an Ihrem Krankenhaus Ärzt*innen aus anderen Abteilungen, in denen Intensivmedizin Teil der Weiterbildung ist, in die Intensivmedizin verschoben?  ⭘ Ja  ⭘ Nein  ⭘ Keine Angaben möglich | At your hospital, have physicians been shifted to critical care from other departments where intensive care is part of their training?  ⭘ Yes  ⭘ No  ⭘ Not specified |
|  | 19a | Falls ja: Re-Allokation von Ärzt*innen aus anderen Abteilungen, in denen Intensivmedizin Teil der Weiterbildung ist, auf die Intensivstation  *Wenn keine genauen Zahlen vorhanden sind, reichen Schätzungen.*  ⭘ Wie viele VK maximal? ___  ⭘ Wie viele Mitarbeiter*innen maximal? ___  ⭘ Wie lange? (in Wochen) ___  ⭘ Keine Angaben möglich | If yes: Reallocation of physicians from other departments, in which intensive care is part of the training, to the intensive care unit  *If no detailed data are available, please estimate.*  ⭘ How many FTP maximum?  ⭘ How many employees maximum? ___  ⭘ How long? (in weeks) ___  ⭘ Not specified |
|  | 20 | Wurden an Ihrem Krankenhaus Ärzt*innen aus anderen Abteilungen, in denen Intensivmedizin NICHT Teil der Weiterbildung ist, in die Intensivmedizin verschoben?  ⭘ Ja  ⭘ Nein  ⭘ Keine Angaben möglich | At your hospital, have physicians been shifted to intensive care from other departments where intensive care is NOT part of their training?  ⭘ Yes  ⭘ No  ⭘ Not specified |
|  | 20a | Falls ja: Re-Allokation von Ärzt*innen aus anderen Abteilungen, in denen Intensivmedizin NICHT Teil der Weiterbildung ist, auf die Intensivstation  *Wenn keine genauen Zahlen vorhanden sind, reichen Schätzungen.*  ⭘ Wie viele VK maximal? ___  ⭘ Wie viele Mitarbeiter*innen maximal? ___  ⭘ Wie lange? (in Wochen) ___  ⭘ Keine Angaben möglich | If yes: Reallocation of physicians from other departments, where intensive care medicine is NOT part of the training, to the intensive care unit  *If no detailed data are available, please estimate.*  ⭘ How many FTP maximum?  ⭘ How many employees maximum? ___  ⭘ How long? (in weeks) ___  ⭘ Not specified |
|  | 21 | Wurden an Ihrem Krankenhaus OP-Pflegekräfte oder OTAs in die Intensivmedizin verschoben?  ⭘ Ja  ⭘ Nein  ⭘ Keine Angaben möglich | Have surgical nurses or surgical technicians been shifted to critical care at your hospital?  ⭘ Yes  ⭘ No  ⭘ Not specified |
|  | 21a | Falls ja: Re-Allokation von OP-Pflegekräften oder OTAs auf die Intensivmedizin  *Wenn keine genauen Zahlen vorhanden sind, reichen Schätzungen.*  ⭘ Wie viele VK maximal? ___  ⭘ Wie viele Mitarbeiter*innen maximal? ___  ⭘ Wie lange? (in Wochen) ___  ⭘ Keine Angaben möglich | If yes: Reallocation of surgical nurses or surgical technicians to the intensive care unit  *If no detailed data are available, please estimate.*  ⭘ How many FTP maximum?  ⭘ How many employees maximum? ___  ⭘ How long? (in weeks) ___  ⭘ Not specified |
|  | 22 | Wurden an Ihrem Krankenhaus Anästhesie-Pflegekräfte oder ATAs in die Intensivmedizin verschoben?  ⭘ Ja  ⭘ Nein  ⭘ Keine Angaben möglich | Have anesthesia nurses or anesthesia technicians been shifted to critical care at your hospital?  ⭘ Yes  ⭘ No  ⭘ Not specified |
|  | 22a | Falls ja: Re-Allokation von Anästhesie-Pflegekräften oder ATAs auf die Intensivmedizin  *Wenn keine genauen Zahlen vorhanden sind, reichen Schätzungen.*  ⭘ Wie viele VK maximal? ___  ⭘ Wie viele Mitarbeiter*innen maximal? ___  ⭘ Wie lange? (in Wochen) ___  ⭘ Keine Angaben möglich | If yes: Reallocation of anesthesia nurses or anesthesia technicians to the intensive care unit  *If no detailed data are available, please estimate.*  ⭘ How many FTP maximum?  ⭘ How many employees maximum? ___  ⭘ How long? (in weeks) ___  ⭘ Not specified |
|  | 23 | Wurden an Ihrem Krankenhaus Pflegekräfte von Normalstation oder Personal aus Funktionsbereichen (wie z. B. Herzkatheter) in die Intensivmedizin verschoben?  ⭘ Ja  ⭘ Nein  ⭘ Keine Angabe möglich | At your hospital, have nurses been moved from normal wards or staff from intervention units (such as cardiac catheterization lab) to critical care?  ⭘ Yes  ⭘ No  ⭘ Not specified |
|  | 23a | Falls ja: Re-Allokation von Pflegekräften von Normalstation oder Personal aus Funktionsbereichen (wie z. B.  Herzkatheter) auf die Intensivmedizin  *Wenn keine genauen Zahlen vorhanden sind, reichen Schätzungen.*  ⭘ Wie viele VK maximal? ___  ⭘ Wie viele Mitarbeiter*innen maximal? ___  ⭘ Wie lange? (in Wochen) ___  ⭘ Keine Angaben möglich | If yes: Reallocation of nurses from normal wards or staff from intervention units (such as cardia catheterization lab) to the intensive care unit  *If no detailed data are available, please estimate.*  ⭘ How many FTP maximum?  ⭘ How many employees maximum? ___  ⭘ How long? (in weeks) ___  ⭘ Not specified |
|  | 24 | Wurden an Ihrem Krankenhaus andere Mitarbeiter*innen in die Intensivmedizin verschoben?  ⭘ Ja  ⭘ Nein  ⭘ Keine Angabe möglich | Have other employees been shifted to critical care at your hospital?  ⭘ Yes  ⭘ No  ⭘ Not specified |
|  | 24a | Falls ja: Re-Allokation andere Mitarbeiter*innen auf die Intensivmedizin  *Wenn keine genauen Zahlen vorhanden sind, reichen Schätzungen.*  ⭘ Wie viele VK maximal? ___  ⭘ Wie viele Mitarbeiter*innen maximal? ___  ⭘ Wie lange? (in Wochen) ___  ⭘ Keine Angaben möglich | If yes: Reallocation of other employees to the intensive care unit  *If no detailed data are available, please estimate.*  ⭘ How many FTP maximum?  ⭘ How many employees maximum? ___  ⭘ How long? (in weeks) ___  ⭘ Not specified |

Abbreviations used: VK *Vollkraftstellen*, FTP *full-time positions*, ICU *intensive care unit,* OTAs *Operationstechnische Assistent*innen,* ATAs *Anästhesietechnische Assistent*innen*
